# Atypical Choroid Plexus Papilloma of the Fourth Ventricle in an Adult: A Case Report

**DOI:** 10.7759/cureus.25256

**Published:** 2022-05-23

**Authors:** Keshav Goel, Uday Birdi, Simon Menaker, Serguei I Bannykh, Chirag Patil

**Affiliations:** 1 Department of Neurosurgery, University of California Los Angeles David Geffen School of Medicine, Los Angeles, USA; 2 Department of Neurosurgery, Cedars-Sinai Medical Center, Maxine Dunitz Neurosurgical Institute, Los Angeles, USA; 3 Department of Pathology & Laboratory Medicine, Cedars-Sinai Medical Center, Los Angeles, USA

**Keywords:** oncogenic mutations, telovelar approach, next generation sequencing, fourth ventricle, atypical choroid plexus papilloma

## Abstract

Atypical choroid plexus papilloma (aCPP) is very rarely seen in adults. Here, we present the case of a 47-year-old male with several months of headache, nausea, dizziness, and imbalance who was found to have an enhancing mass of the fourth ventricle with imaging findings suggestive of likely ependymoma. The patient underwent suboccipital craniotomy with C1 laminectomy and telovelar approach for gross-total resection of the lesion, with final pathology demonstrating WHO grade II aCPP. Subsequent genomic analysis showed a biologically relevant TERT mutation, as well as several variants of unknown significance. We conclude that aCPP is a rare, benign entity diagnosed by tissue sample that is potentially curative with surgical resection and may harbor targetable genetic mutations.

## Introduction

Choroid plexus papilloma (CPP) is a rare benign neoplasm of the choroid plexus, accounting for less than 1% of all intracranial neoplasms in adults. Depending on location, the clinical presentation of CPP may include signs/symptoms of obstructive hydrocephalus, vertigo, diplopia, lateral gaze palsies, and visual field defects [1]. CPPs with histopathological features suggestive of higher mitotic activity but without hallmarks of malignancy are classified as atypical (aCPP). Adult aCPPs are very rare tumors typically localized to the fourth ventricle [2]. Maximal safe resection is the primary treatment and gross-total resection (GTR) is often curative for grade I lesions with low recurrence rates [3].

Here, we present the case of a fourth ventricular aCPP in an adult patient. We discuss the pertinent clinical, operative, and histopathological findings, as well as the results and implications of associated next-generation sequencing (NGS).

## Case presentation

**Figure 1:**
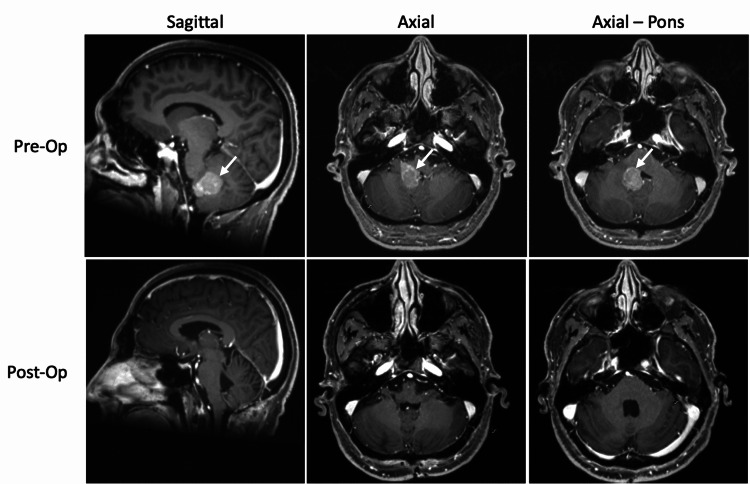
Pre- and postoperative T1-weighted gadolinium-enhanced MRI images in sagittal and axial planes. Preoperative T1-weighted MRI reveals a 2.6 cm enhancing fourth ventricular mass (white arrows), with extension to the right foramen of Luschka. There is a mass effect on the ipsilateral medulla without evidence of obstructive hydrocephalus. Postoperative MRI demonstrates image complete, gross-total resection of the tumor.

A 47-year-old male presented with a 2-month history of head pressure, nausea, dizziness, and tandem gait imbalance. The review of symptoms was negative for diplopia, sensory abnormalities, and limb weakness. Magnetic resonance imaging (MRI) demonstrated a 2.6 cm well-circumscribed contrast-enhancing mass of the fourth ventricle with extension into the right foramen of Luschka without radiographic evidence of hydrocephalus (Figure 1). Spinal MRI was negative for drop metastases.

The patient underwent suboccipital craniotomy and C1 laminectomy with subsequent telovelar approach to the fourth ventricle for tumor resection. The right-eccentric tumor was dissected off the floor of the fourth ventricle, cerebellar peduncle, and the tela. The tumor was debulked and followed laterally all the way to the foramen of Luschka. The cerebellopontine angle with cranial nerve IX was visualized at the end of the resection through the expanded foramen of Luschka. The tumor was quite vascular and had significant areas of attachment to the lateral border of the brainstem, tela, and right cerebellar peduncle. The frozen section returned as likely ependymoma.

Postoperative imaging showed complete resection of the tumor (Figure 1). Immediate postoperative complications included dysconjugate gaze, diplopia, right-sided hearing loss, and difficulty with coordination. The patient’s recovery was also complicated by hospital-acquired pneumonia.

Histopathology

**Figure 2:**
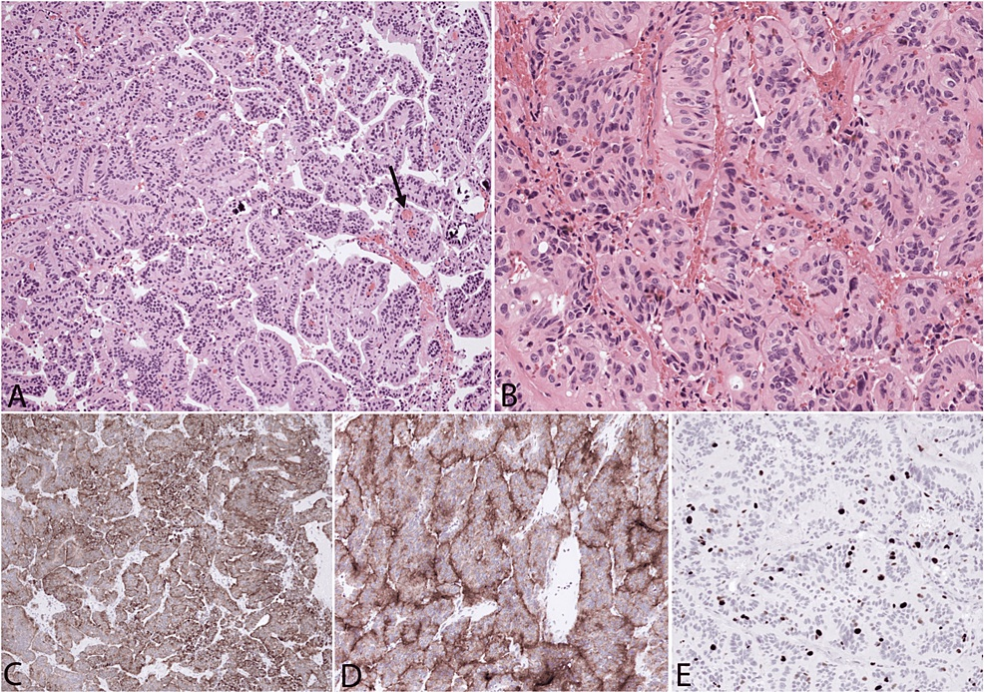
Histologic finding. Hematoxylin and eosin-stained sections (A & B) show papillary growth patterns with psammoma bodies (black arrow) (A) characteristic of a well-differentiated choroid plexus papilloma, but also extensive foci of acinar architecture with substantial nuclear pleomorphism (white arrow) (B). The tumor is positive for low molecular weight cytokeratin (C), and synaptophysin (D) and shows a slightly elevated Ki-67 proliferative index (E). Original magnifications: 100× (A, C-E), 200× (B).

Histopathology on permanent sections was discordant with intraoperative pathology. The lesion showed true papillae with entrapped psammoma bodies characteristic of CPP (Figure 2A). In addition, perivascular arrangements, as well as acinar growth patterns of pleomorphic cells, were seen (Figure 2B) accompanied by scattered apoptotic bodies and small foci of tumor necrosis. There was no evidence of brain invasion and mitoses were not apparent, but the Ki-67 proliferative index was 4% (Figure 2E). The tumor showed strong staining for low molecular weight keratin (CAM 5.2) (Figure 2C), epithelial membrane antigen, E-cadherin, S-100, and synaptophysin (Figure 2D). Scattered cells were positive for glial fibrillary acidic protein, transthyretin, cytokeratin (CK) 7, and p53. OLIG-2, CK-20, and high molecular weight CK were negative. Growth patterns and elevated Ki-67 index allowed us to render a diagnosis of atypical CPP, WHO, grade II.

Tempus NGS revealed a biologically relevant TERT promoter mutation (c.-124C>T) with a tumor mutational burden in the 37th percentile and stable microsatellites. Mutational variants of unknown significance included KMT2D, UGP2, FCGR2A, and ZFHX3.

The patient remains recurrence-free as of his last one-year postoperative follow-up visit. He is able to ambulate normally and is back to work full time. His diplopia has also completely resolved. He continues to have right-sided hearing loss.

## Discussion

Surgical approach

Fourth ventricular tumors like this reported aCPP are typically accessed via a suboccipital craniotomy and either transvermian or telovelar approaches. In addition, certain case studies (e.g. Lee et al.) report the usage of transcortical approaches [4]. Turkoglu et al. reported the use of a telovelar approach in nine patients with choroid plexus tumors in the fourth ventricle. Satisfactory postoperative outcomes were achieved in 8/9 patients, while aCPPs and CPPs remained progression-free until the last follow-up [5]. In a study of 40 patients presenting with various fourth ventricular masses with a 1:1 distribution of telovelar vs. transvermian resection, 75% of telovelar cases and 60% of transvermian cases achieved GTR. In addition, postoperative complications were higher with the transvermian approach, most notably cerebellar mutism and bulbar palsy, each with an incidence of 15% in transvermian and no incidence in telovelar cases [6]. The telovelar approach is currently preferred over the transvermian approach, which has a higher risk of cerebellar mutism. Anatomic studies have shown better vertical access from the obex to the cerebral aqueduct as well as superior lateral access to the foramen of Luschka with the telovelar approach compared to the transvermian approach. In summary, due to the larger plane of access and avoidance of cerebellar vermis disruption, the telovelar approach provides multiple benefits over the traditional transvermian approach for the management of fourth ventricular tumors [7].

Histology

According to the WHO, CPPs are graded from I to III based on specific histological criteria, with benign CPPs classified as grade I and malignant choroid plexus carcinomas (CPCs) as grade III. Atypical CPPs, first recognized as a distinct entity in 2007 and classified as grade II, are intermediary tumors that exhibit characteristics of both benign CPPs and CPCs [8]. Histologically, aCPPs typically exhibit increased mitotic factors defined as greater than two mitoses per 10 high-powered fields, undefined papillary patterns, and moderate nuclear polymorphisms [9]. Although the number of mitotic bodies observed in our patient was below this threshold, his tumor was classified as grade II given the presence of pleomorphic nuclei with perivascular arrangements of nuclear polymorphisms, as well as foci of necrosis, without hallmark features of malignancy [5]. Of note, Jeibmann et al. reported the recurrence rate of aCPPs as 29%, compared to 6% in benign grade I lesions, with increased mitotic activity a predictor for recurrence [4,10].

Genetic analysis

The Tempus xT assay is an oncological testing panel that utilizes NGS to detect genomic alterations in 648 genes and aid in the selection of targeted therapies. It reveals both genetic variations of biological relevance, which produce functionally significant genetic alterations, and variants of unknown significance, whose alterations have an unclear effect on oncogenesis. Our patient’s assay revealed five mutations of unknown significance, namely KMT2D, UGP2, FCGR2A, and two mutations of ZFHX3 in a splice region. In addition, there was one biologically relevant mutation in TERT.

Understanding the function of these genes allows for extrapolation regarding their potential contribution to features of our patient’s tumor. The growth of the aCPP might, in part, be attributed to a mutation in the KMT2D gene, which encodes a histone methyltransferase that is part of a protein complex regulating the transcription of estrogen receptor genes. KMT2D has been reported as an essential mutation for cancer cell survival, as its depletion improves chemotherapeutic sensitivity [11]. UGP2 codes for an essential enzyme in nucleotide sugar metabolism and is responsible for catalyzing the conversion of glucose-1-phosphate to uridine diphosphate (UDP)-glucose. Oncogenic mechanisms involving UGP2 gain-of-function mutations result in higher intracellular glycogen levels and overactivation of estimated glomerular filtration rate (EGFR) targets via N-acetylation [12]. The FCGR2A gene is part of a family of immunoglobulin Fc receptor genes, which encodes a cell surface receptor found on phagocytic cells. The FCGR2A mutation identified in our patient’s tumor might therefore have contributed to the foci of tissue necrosis seen in pathology, a critical component in its classification as an atypical lesion [10]. Finally, ZFHX3 encodes a protein that regulates neuronal differentiation and tumor suppression, a mutation of which might have, in part, facilitated unregulated and aberrantly differentiated cell growth in this case [13].

Mutation in the promoter region of the TERT gene leads to overexpression of telomerase in 85-90% of human malignancies [14]. Activation of TERT has been shown to increase proliferation and resist apoptosis [15]. Furthermore, TERT mutations are highly associated with central nervous system (CNS) tumors, and in particular with unfavorable outcomes in choroid plexus tumors. Currently, there are no direct TERT inhibitors, but drugs targeting the TERT pathway are under investigation [16]. Each of the mutations described above represents a potential pathogenic mechanism and a commensurate therapeutic target. Thus, the Tempus xT assay and NGS may ultimately allow for personalized therapeutic approaches to treat rare tumors like aCPPs, especially in cases of tumor recurrence after initial surgical resection.

Beyond personalized treatment strategies, other newer treatment paradigms exist which may be considered in patients with aCPPs. Immunotherapeutic agents, in particular immune checkpoint inhibitors, now play an integral role in the medical management of a variety of different cancers including lung cancer, renal cell carcinoma, and melanoma, and their potential benefit in CNS malignancies is being widely studied [17]. This is, in part, based on recent studies elucidating the lymphatic architecture of the brain, which demonstrate that T cells traffic via meningeal blood vessels into CSF spaces and ultimately into the brain parenchyma via the choroid plexus [18,19]. Therefore, there is the potential for T cell-mediated therapies to play a role in the management of CNS tumors like choroid plexus malignancies. Although immunotherapy is not yet the standard of care, further investigation is underway, notably a phase II trial studying the effect of nivolumab, a programmed death (PD-1) inhibitor, in the treatment of recurrent select rare CNS cancers including aCPPs (NCT03173950) [20].

## Conclusions

This work presents a 47-year-old male patient presenting with a history of head pressure, nausea, dizziness, and tandem gait imbalance. Preoperative imaging demonstrated a 2.6 cm mass of the fourth ventricle with hydrocephalus, which was characterized as an atypical CPP on histological analysis and a TERT mutation on Tempus NGS. The patient underwent suboccipital craniotomy with C1 laminectomy, followed by a telovelar approach to the fourth ventricle to achieve GTR. Minimal postoperative complications were noted and compared to traditional approaches such as transvermian, strengthening the potential utility of the telovelar surgical approach.

This case report also demonstrates the use of next-generation DNA sequencing to better understand the tumor’s genetic profile. Doing so allows for targeted molecular therapies to be used and improve precision-based approaches to intracranial tumor management. Finally, this report also exemplifies the importance of comprehensive histopathological examination of tissue specimens for accurate diagnosis.
